# Constitutive accessibility of circulating proteins to hippocampal neurons in physiologically normal rats

**DOI:** 10.1002/brb3.1544

**Published:** 2020-01-27

**Authors:** Sawako Hamasaki, Takao Mukuda, Yuka Koyama, Hironobu Nakane, Toshiyuki Kaidoh

**Affiliations:** ^1^ Department of Anatomy Faculty of Medicine Tottori University Yonago Japan

**Keywords:** albumin, blood–brain barrier, Evans blue dye, granule cell layer, hilus

## Abstract

**Introduction:**

Although the hippocampus (HIP) is thought impermeable to blood‐borne proteins because of the integrity of the blood–brain barrier (BBB), it was recently suggested to be susceptible to hydrophilic hormones. The present study determined the accessibility of blood‐borne signal molecules such as hormones to hippocampal neurons in physiologically normal rats.

**Methods:**

As a probe for accessibility, Evans blue dye (EB) that rapidly binds to albumin (Alb), which is impermeable to the BBB, was injected intravenously. To increase the vascular permeability of the BBB, a daily single administration of angiotensin II (Ang II) was applied intravenously for seven consecutive days.

**Results:**

Fifteen minutes after the injection of EB, histological observation revealed that a number of neurons had entrapped and accumulated EB into their cell bodies in the hippocampal dentate gyrus in all rats. Of these, relatively large oval neurons (>15 µm) in the hilus and molecular layer showed parvalbumin immunopositivity, indicating they are GABAergic interneurons. The population of EB‐accumulating neurons (approximately 10 µm) were localized in the inner margin of the granule cell layer, suggesting they were granule cells. However, the number of EB‐positive neurons did not change in rats treated with Ang II compared with vehicle injection.

**Conclusions:**

These findings suggest an intriguing possibility that blood‐derived proteins such as hormones have access to hippocampal neurons constitutively in the absence of stimuli that increase the vascular permeability of the BBB in a physiologically normal state.

## Graphical abstract

An albumin‐binding dye Evans blue (EB) was injected intravenously in normal rats; then, neurons of the hippocampal dentate gyrus (HIP) rapidly entrapped the dye, as in the arcuate nucleus (Arc). This suggests that circulating proteins may be accessible to the hippocampus constitutively to act on neurons directly.

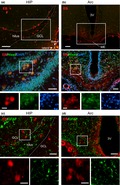

## 1 INTRODUCTION

Almost all brain regions, including the hippocampus (HIP), are thought to be isolated from the systemic circulation by the blood–brain barrier (BBB), which provides tight control over the passage of small molecules from the blood. Furthermore, BBB integrity prohibits leakage of circulating proteins such as albumin (Alb) and hydrophilic hormones, in the absence of controlled transendothelial transport systems that facilitate transcytosis, such as insulin‐like growth factor 1. However, circumventricular organs (CVOs) including the subfornical organ (SFO) and median eminence (ME), which possess fenestrated capillaries and lack a BBB, are widely accepted as providing free access for hormones to neurons localized within and around the CVOs.

Various physiologically abnormal conditions including chronic hypertension and traumatic injury frequently disrupt the BBB in the HIP to increase vascular permeability, suggesting the selective vulnerability of the BBB in the HIP compared with other brain regions (Ueno et al., 2004; del Valle, Camins, Pallàs, Vilaplana, & Pelegrí, 2008). Although the underlying mechanisms involved in the disintegration of the BBB under these conditions are poorly understood, an octapeptide, angiotensin II (Ang II), was demonstrated to be critical for the modulation of hippocampal BBB permeability in vivo (Biancardi, Son, Ahmadi, Filosa, & Stern, 2014; Li et al., 2014, 2016). Furthermore, an in vitro study using BBB microvascular endothelial cell culture showed that Ang II led to structural changes including pore induction on endothelial cells, which increased BBB permeability (Fleegal‐DeMotta, Doghu, & Banks, 2009).

Contrary to the established theory of BBB integrity, accumulating evidence has suggested that several blood‐derived products can penetrate into the HIP in the absence of stimuli that increase BBB permeability under healthy conditions. Ueno and colleagues assessed potential penetration into the HIP from the blood by tracing an exogenous probe, horseradish peroxidase (HRP), injected intravenously into normal mice, which leaks into the SFO and then diffuses to the HIP (Ueno et al., 1994, 1997, 2000). In addition, circulating ghrelin, a 28‐residue peptide hormone, was translocated from blood to the HIP at a low level (Banks, Tschöp, Robinson, & Heiman, 2002; Rhea et al., 2018). This peptide regulates the synapse density of the HIP (Diano et al., 2006) and stimulates adult neurogenesis directly (Li et al., 2013). Recently, we found that a transient increase in plasma Ang II levels triggered enhanced neurogenesis in the adult rat HIP, probably through trophic factor production in hippocampal astrocytes on which the peptide acts directly (Koyama, Mukuda, Hamasaki, Nakane, & Kaidoh, 2018; Mukuda, Koyama, Hamasaki, Kaidoh, & Furukawa, 2014). However, direct evidence that blood‐borne products can access hippocampal neurons constitutively has not been reported.

The fluorescent dye Evans blue (EB) is a useful probe to explore leaky blood vessels and deficits of the BBB because it rapidly binds to a plasma carrier protein Alb which cannot cross the BBB (Yao, Xue, Yu, Ni, & Chen, 2018). In addition to the CVOs, a large amount of EB‐Alb complex extravasation, induced by pathological conditions such as traumatic brain injury and inflammation, was detected in brain parenchyma as a blue stain macroscopically or as an intense red signal in the extracellular space of brain parenchyma by fluorescent microscopy (Alonso, Reinz, Fatar, Hennerici, & Meairs, 2011; Rákos et al., 2007; Roe et al., 2012; Yang et al., 2007). However, when there is a minimal penetration of Alb into the brain parenchyma, it is extremely hard to distinguish low EB signals using conventional anatomical and histological examination because of the poor signal‐to‐noise (S/N) ratio. However, EB is entrapped by neurons (Albanese & Bentivoglio, 1982; Mukuda & Ando, 2003, 2010) and the intracellular accumulation of EB following neuronal uptake intensifies the signal, thereby allowing minimal amounts of EB to be measured, and the direct accessibility of blood‐borne products including hormones to hippocampal neurons can be assessed.

The aim of the present study was to examine the direct accessibility of blood‐borne proteins to hippocampal neurons in physiologically normal rats using EB, which binds to Alb in the blood. An intravenous injection of EB was made 15 min before sacrifice. In addition, to determine changes in the accessibility of EB, a daily single dose of Ang II, to increase BBB permeability, was given for seven consecutive days. Neuronal uptake of EB was observed in brain sections by fluorescent microscopy. The number of EB‐positive neurons was counted and compared between rats administered vehicle or Ang II. Furthermore, immunohistochemical staining was performed on EB‐accumulating neurons to identify their neurochemical features.

## 2 MATERIALS AND METHODS

### 2.1 Animals

All animal procedures in the present study were performed in accordance with the Guidelines for the Care and Use of Laboratory Animals of Tottori University and Guidelines for Proper Conduct of Animal Experiments decreed by the Science Council and Ministry of Education, Culture, Sports, Science and Technology of Japan. All experimental protocols were approved by Tottori University Institutional Animal Care and Use Committee (approved protocol numbers: 17‐Y‐14).

Male Long‐Evans rats (7–9 weeks old) used in the present study were bred in our laboratory. All rats were housed under the standard conditions of a 12‐hr/12‐hr light/dark cycle at 23–25°C and received food and water ad libitum. Until the experiments began, 3–4 rats were housed in each cage.

### 2.2 Surgical procedures

To administer various substances, all rats were implanted with catheters into the right atrium via the right jugular vein. Briefly, rats were anesthetized by an intraperitoneal injection of thiamylal sodium (60 mg/kg, Nichi‐Iko Pharmaceutical). After shaving the hair of the right chest and dorsal neck, the rat was placed ventral side up and a small incision was made in the right supraclavicular skin to provide access to the right jugular vein. The jugular vein was isolated from the subcutaneous fatty tissue and a small cut was made, through which a polyurethane tube (1.02 mm outer diameter, MRE‐040, Eicom) primed with heparinized saline (10‐U heparin sodium in 1 ml 0.9% NaCl) was inserted toward the right atrium. The catheter was secured in the blood vessel with cotton threads. The distal tip of the catheter was capped and passed through the cervical subcutaneous tissue toward the back and exteriorized through a small incision on the dorsal neck skin. The incisions were sutured with cotton threads and wiped clean. After surgery, rats were housed individually with fresh bedding for at least 3 days for recovery. The catheter implanted into the rat was flushed daily with heparinized saline (<50 µl) to prevent it from clogging.

### 2.3 Administration of Ang II, EB, and 5‐bromo‐2′‐deoxyuridine into the systemic circulation

Ang II was reported to increase the permeability of the BBB and cause leakage of blood‐derived large molecules (Biancardi et al., 2014; Fleegal‐DeMotta et al., 2009). To induce this leakage, a single dose of Ang II (200 µl, 10^–5^ M in vehicle: 10‐U heparin sodium in 1 ml 0.9% NaCl) was given to rats via the catheter, followed by vehicle (100 µl) to flush out Ang II in the catheter (*n* = 7). This was repeated daily during the experimental period consisting of seven consecutive days. In our preliminary study, we observed the pharmacological effect of a single injection of Ang II, whereby the arterial blood pressure was increased transiently. In addition, this injection increased the number of new cells and immature neurons in the HIP, indicating it was effective in the HIP (Mukuda et al., 2014). For controls, 300 µl of vehicle was administered daily (*n* = 6). To evaluate the physiological effect of the increased plasma levels of Ang II, the volume of water intake over a 15‐min period was measured after the intravascular injection. An increase in plasma Ang II is well established to elicit potent thirst and compel water drinking (Fitzsimons, 1998).

Evans blue binds to Alb immediately and forms a complex when the dye enters the blood, and this EB‐Alb complex cannot cross an intact BBB (Yao et al., 2018). Therefore, EB is widely used to assess the BBB integrity in the brain, in which extravasation of EB into the brain parenchyma is a good probe for identifying regions where the BBB is leaky (Alonso et al., 2011; Rákos et al., 2007; Uyama et al., 1988). In addition, EB is often used to trace neuronal innervation because it is taken up intracellularly by various types of living neurons in the peripheral and central tissues, and accumulates in their somata (Mukuda & Ando, 2003, 2010). To detect the extravasation of EB‐Alb complex into the HIP, EB (200 µl, 20 mg/ml in vehicle) was injected via the catheter 15 min before transcardial perfusion on the last day followed by 100 µl of vehicle to flush out the remaining EB. To label proliferating cells in the HIP, 5‐bromo‐2′‐deoxyuridine (BrdU, 200 µl, 10 mg/kg in vehicle) was given via the catheter on days 4–7 of the experimental period.

### 2.4 Tissue preparation

Fifteen min after EB injection, rats were deeply anesthetized by an injection of excess thiamylal sodium and perfused transcardially with heparinized phosphate‐buffered saline (PBS, pH 7.4), followed by 4% paraformaldehyde (PFA) in 0.1 M phosphate buffer (PB, pH 7.4). The brain was isolated and postfixed in 4% PFA in 0.1 M PB for 48 hr at 4°C, cryoprotected in 30% sucrose in 0.1 M PB, and embedded in OCT Compound (Sakura). Serial samples were cut coronally at a thickness of 20 µm using a frozen microtome (CM1520, Leica), and the sections were mounted onto adhesive glass slides (S9904, Matsunami).

### 2.5 Histology for EB and quantification

In a preliminary examination, washing of tissue sections in the histological procedures reduced the presence of EB in the extracellular space or neurons. To avoid loss of the dye, a histological examination of EB was performed using brain sections immediately after cutting without washing or coverslipping. Fluorescent images were observed and captured using a fluorescent microscope (BX 51, Olympus) equipped with a digital full‐color camera (DP73, Olympus). Specific fluorescence of EB (excitation peak at 470–540 nm, emission peak at 680 nm) was emitted (emission wavelength of >575 nm) by illuminating the section with green light (530–550 nm). Exposure time was adjusted to avoid saturation and photobleaching. For each experiment, the same optimized conditions were applied to the series of samples. Images were analyzed using cellSens software (Olympus). After finishing EB examination, identical sections were used for the following staining.

Evans blue was quantified to assess the amount of leakage from the blood. A large amount of leakage of EB can be quantified by measuring the intensity of fluorescence emitted from dye entrapped in the extracellular space. However, it is difficult to distinguish low signals of EB by conventional analysis because of the poor S/N ratio. To overcome this, the leakage of EB was determined by counting the number of distinctly distinguishable neurons (>15 µm in diameter or long axis) that had entrapped EB in the granule cell layer (GCL) and hilus using fluorescent images. EB‐positive cells (<15 µm) in the GCL were not counted because the precise isolation of each cell was difficult to determine because of their close localization. Counting was performed using every fourth section of the left hemisphere of the rostral part of the HIP from the rostral tip to 1,200 µm caudal (15 sections with 80‐µm intervals) in each animal. Then, the count was multiplied by 4, thereby estimating the number of EB‐positive cells in the rostral HIP of each animal. Values are expressed as the means ± standard deviation (*SD*). Statistical significance between animals given Ang II and vehicle was determined using the unpaired *t* test. *p* < .05 was considered statistically significant.

### 2.6 Histochemistry and immunohistochemistry

All sections for histochemical or immunohistochemical analysis, except for Alb immunostaining, were pretreated with blocking solution (5% normal donkey or goat serum, 0.1% Triton X‐100, 0.05% Tween‐20 in PBS) for 1 hr before incubation with a histochemical probe or primary antibodies. All histological procedures were performed at room temperature unless otherwise stated. To determine albumin extravasation into the brain, sections were pretreated with a diluent containing 5% normal donkey serum in PBS and then incubated with sheep polyclonal anti‐Alb antibody (1:200, A110‐134A, Bethyl), diluted with the diluent, overnight at 4°C. To identify proliferating cells using BrdU, sections were treated with proteinase K (0.02 mg/ml in PBS, P6566, Sigma) for 10 min. After rinsing with PBS, the sections were incubated with 2 N HCl for 30 min at 37°C and then with 0.1 M boric acid (pH 8.2) for 10 min. After rinsing with PBS and blocking, the sections were incubated with mouse monoclonal anti‐BrdU antibody (1:500, MAB4072, Millipore), diluted with blocking solution, overnight at 4°C. To determine the types of cells, sections were incubated for 48 hr at 4°C with an appropriate combination of the following primary antibodies diluted with blocking solution: goat polyclonal antibody for doublecortin (DCX, 1:250, sc‐8066, Santa Cruz Biotechnology) for immature neurons, rabbit polyclonal antibody for parvalbumin (PV, 1:500, GTX 11,427, GeneTex) for GABAergic interneurons, and goat polyclonal antibody for glial fibrillary acidic protein (GFAP, 1:1,000, ab53554, Abcam) for astrocytes.

Sections incubated with primary antibodies were rinsed with PBS and then incubated with an appropriate combination of the following secondary antibodies conjugated with fluorophores together with green fluorescent Nissl (1:500, N‐21480, Molecular Probes) or 4′,6‐diamidino‐2‐phenylindole dilactate (1:1,000, DAPI, D9564, Sigma), diluted in blocking solution, for 2 hr. The secondary antibodies used were as follows: donkey anti‐sheep IgG antibody conjugated with Alexa Fluor 568 (1:500, ab175712, Abcam), donkey anti‐goat IgG antibody conjugated with AMCA (1:500, 705‐155‐147, Jackson ImmunoResearch) or with Cy3 (1:500, 705‐165‐147, Jackson ImmunoResearch), donkey anti‐rabbit IgG antibody conjugated with DyLight 488 (1:750, 711‐485‐152, Jackson ImmunoResearch), and goat anti‐mouse IgG antibody conjugated with Cy3 (1:500, 115‐165‐003, Jackson ImmunoResearch). After rinsing with PBS, the sections were embedded and coverslipped. Stained preparations were examined under a fluorescence microscope (BX51, Olympus) equipped with a digital full‐color camera (DP73, Olympus). Exposure time was adjusted to avoid saturation and photobleaching. For each experiment, the same optimized conditions were applied to the series of samples. Images were acquired and analyzed using cellSens software (Olympus).

## 3 RESULTS

### 3.1 Extravasation of Alb in the brain

**Figure 1:**
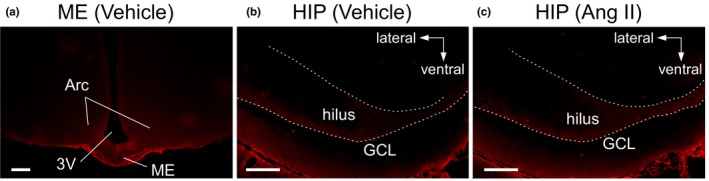
Immunostaining of albumin (Alb) extravasated into the brain. Photomicrographs show the median eminence (ME) (a) and hippocampus (HIP) (b, c). (a, b) Brain tissue slices obtained from a rat given a single injection of vehicle (300 µl). (c) Hippocampal section from a rat injected with Ang II (10^–5^ M, 200 µl plus 100 µl of vehicle). White broken lines on the HIP indicate the hilar border of the granule cell layer (GCL) of the dentate gyrus. Scale bars, 200 µm. Arc, arcuate nucleus; 3V, third ventricle

As a positive control, immunoreactivity of Alb was found in the neuropil of the ME in vehicle‐ and Ang II‐treated rats, which expanded toward the arcuate nucleus of the hypothalamus (Arc) (Figure 1a). In the HIP, albumin signals were undetectable in the neuropil of rats given vehicle (Figure 1b). When Ang II was given daily, low albumin signals were observed along the hilar margin of the GCL and in the hilus in the hippocampal dentate gyrus, but it was difficult to distinguish between them for quantitative analysis because of the poor S/N ratio (Figure 1c).

**Figure 2:**
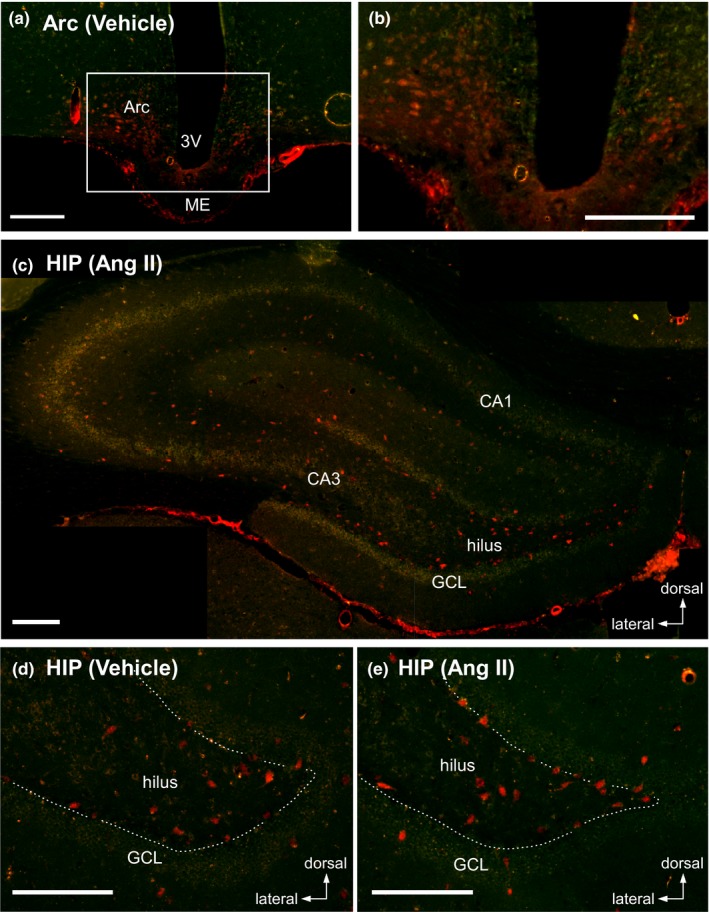
Accumulation of Evans blue dye (EB) in cell bodies in the brain. Rats were given EB (200 µl, 20 mg/ml in vehicle) via an implanted atrial catheter 15 min before sacrifice. (a, b) Photomicrographs show the uptake of EB in cells of the Arc in a rat injected with vehicle. The enclosed area with a white line in (a) is magnified in (b). (c) Low magnification of the HIP obtained from a rat given Ang II. (d, e) High magnification of the hippocampal dentate gyrus from rats given vehicle (d) or Ang II (e). White broken lines on the HIP indicate the hilar border of the GCL of the dentate gyrus. Scale bars, 200 µm

As observed for Alb immunoreactivity, EB signals were observed in the neuropil in the ME‐Arc (Figure 2a). In addition to EB in the neuropil, an intense signal of the dye was found in many cells in the Arc (Figure 2b), indicating that the EB‐Alb complex that had penetrated into the extracellular space through the leaky blood vessels of the ME was taken into cells of the Arc. In the HIP, it was difficult to detect an EB signal in the neuropil in rats given vehicle or treated with Ang II, but intense EB fluorescence was identified in cell bodies in the hippocampal dentate gyrus in both groups (Figure 2c–e). EB‐positive cells were frequently found in the hilus and GCL, while few were observed in the hippocampal formation: No pyramidal neurons in Ammon's horn (CA) had accumulated EB (Figure 2c). In the hippocampal dentate gyrus of both groups, the somata of EB‐positive hilar cells, many of which were localized adjacent to the GCL, were oval or spindle‐like and >15 µm in long axis (Figure 2d,e). In the GCL, a large number of EB‐positive cells were localized in the hilar margin of the GCL (possibly including the subgranular zone, SGZ) and a few in the deep layer: They had a round or polygonal shape and were approximately 10 µm in diameter (Figure 2d,e). In addition, the molecular layer sometimes contained a few EB‐positive cells that were large, round, or oval (>15 µm in diameter or long axis) (Figure 2d,e).

**Figure 3:**
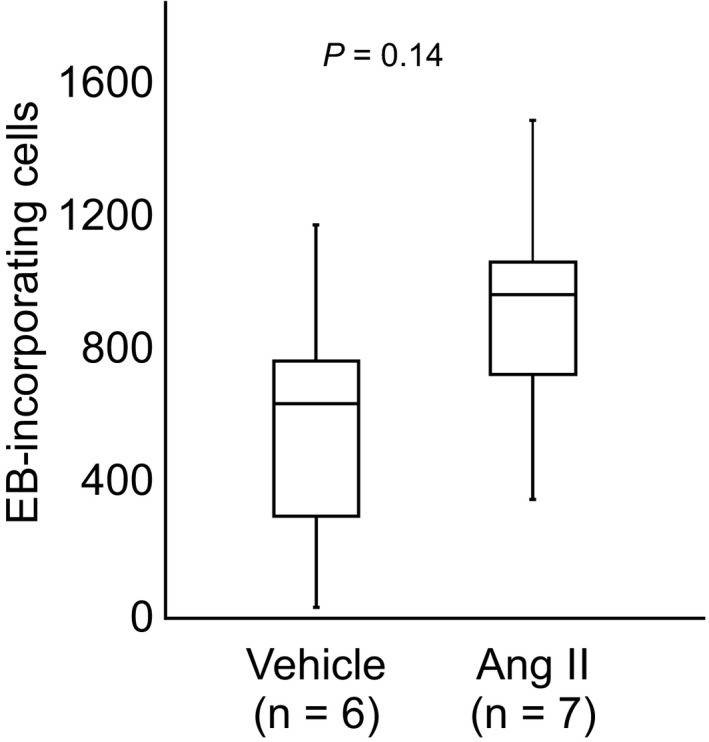
A box‐and‐whisker diagram showing the number of EB‐incorporating cells in the rostral part of the hippocampal dentate gyrus (from the rostral tip to 1,200 µm caudal) in rats given vehicle or Ang II. Statistical comparison was performed using an unpaired *t* test

Evans blue‐positive cells were often found in the rostral region of the dentate gyrus in the HIP and became rare toward the caudal region. The number of hilar EB‐positive cells from the rostral tip to 1,200 µm caudal was slightly increased in rats given Ang II compared with vehicle, although this did not reach statistical significance (583.3 ± 417.4 in vehicle vs. 923.4 ± 137.4 in Ang II, unpaired *t* test, *p* = .14) (Figure 3).

**Figure 4:**
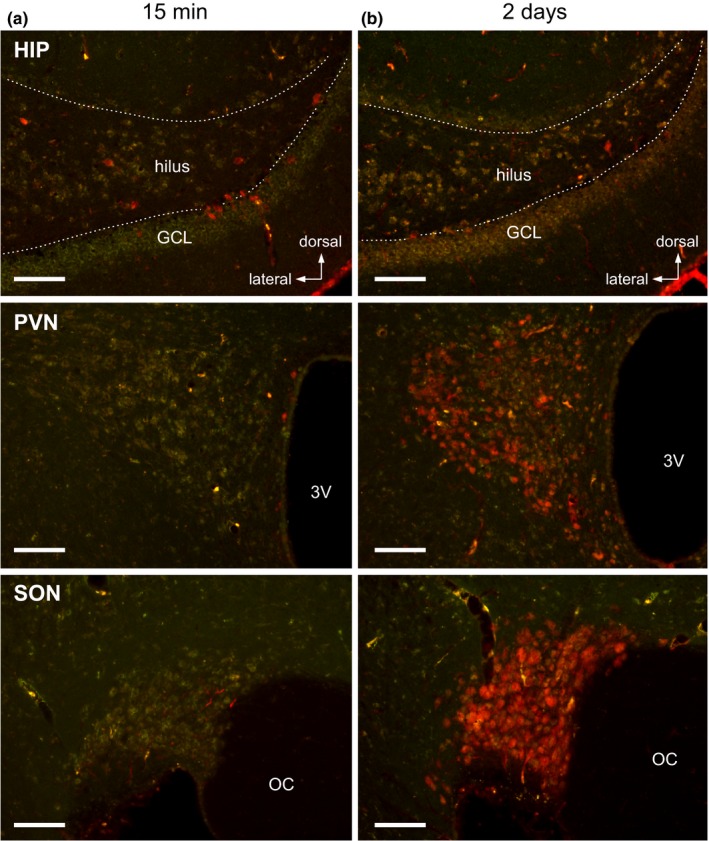
Accumulation of EB in hippocampal and hypothalamic cells at different time points after EB injection. Rats were given EB (200 µl, 20 mg/ml in vehicle) via an implanted atrial catheter 15 min (a) or 2 days (b) before sacrifice. Photomicrographs show the HIP (top), periventricular (PVN) (middle), and supraoptic (SON) (bottom) nuclei, in each left hemisphere. White broken lines in the HIP indicate the hilar border of the GCL. Scale bars, 100 µm. OC, optic chiasma

Evans blue accumulation in hippocampal cells was observed at least 15 min after the intravascular injection. Over the same period, cells in the Arc rapidly entrapped EB. However, EB signals were not detected in neuroendocrine neurons in the paraventricular (PVN) and supraoptic (SON) nuclei (Figure 4a). Given sufficient time for the dye to be transported from axon terminals in contact with blood in the pituitary (e.g., 2 days), the nuclei of all neuroendocrine neurons were well stained with blood‐borne EB (Figure 4b). In addition, hippocampal cells also entrapped EB over this long period. This suggests that EB entrapped by hippocampal cells reached the cells at a sufficiently high concentration to be accumulated in their cell bodies over a short period.

### 3.2 Types of EB‐accumulating cells in the hippocampus

**Figure 5:**
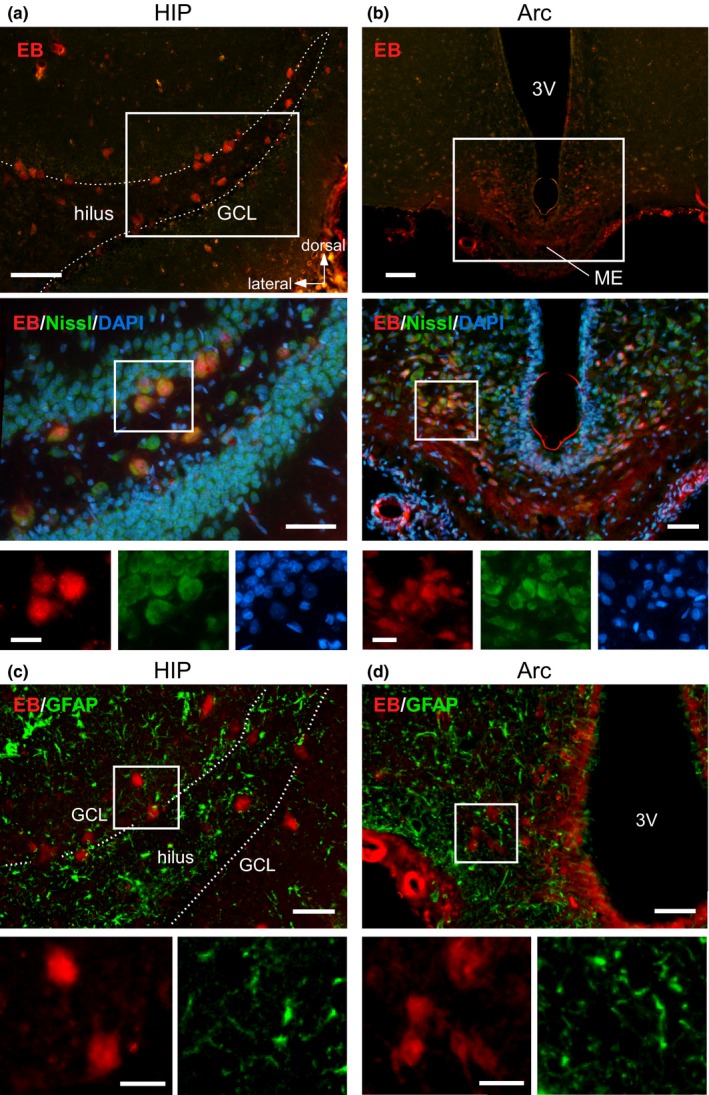
Neural staining in EB‐incorporating cells of the HIP and Arc. (a, b) EB‐incorporating cells were labeled with a green fluorescent Nissl stain in the HIP (a) and Arc (b). Top panels show cells incorporating EB (red). Middle panels show magnified areas enclosed with white squares in the top panels, which are merged with signals of the green fluorescent Nissl stain (green) and DAPI (blue). Bottom panels are the respective color images of EB, Nissl, and DAPI in the area enclosed with white squares in the middle panels. (c, d) EB‐incorporating cells were immunostained for glial fibrillary acidic protein (GFAP) in the HIP (c) and Arc (d). Top panels show GFAP‐immunoreactive astrocytes (green), together with the EB signal. Bottom panels are the respective color images of EB and GFAP in the areas enclosed with white squares in the top panels. White broken lines in the HIP indicate the hilar border of the GCL. Scale bars, 100 µm (top in a, b), 50 µm (middle in a, b and top in c, d), and 20 µm (bottom in a–d)

All EB‐positive cells were colabeled with fluorescent Nissl signals in the hippocampal dentate gyrus (Figure 5a) and Arc (Figure 5b). In contrast, no cell incorporating EB showed immunoreactivity for GFAP (Figure 5c,d). These results indicate that the EB‐positive cells were not astrocytes. Considering the pattern of Nissl staining and the cell morphology including laminar distribution and shape, EB‐positive cells were likely to be neurons.

**Figure 6:**
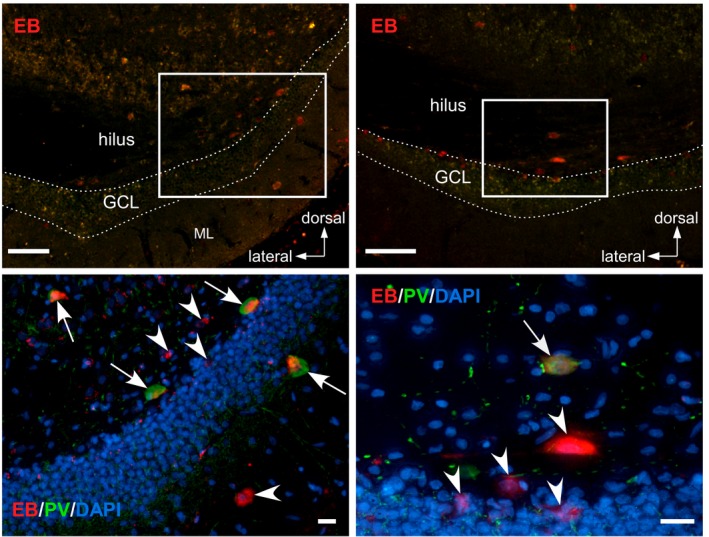
Immunohistochemistry for parvalbumin (PV) in the HIP. Top panels show EB‐incorporating neurons (red). Bottom panels indicate PV immunoreactivity (green) together with EB and DAPI (blue) signals in the areas enclosed with white squares in the top panels. White broken lines indicate the hilar border of the GCL. Arrows indicate neurons double‐positive to EB and PV. Arrowheads indicate neurons positive for EB but negative for PV. Scale bars, 100 µm (top), 20 µm (bottom). ML, molecular layer

In the hilus and molecular layer, some large EB‐positive cells (>15 µm) were immunoreactive for PV (Figure 6), a marker for GABAergic interneurons in the hippocampus (Kosaka, Katsumaru, Hama, Wu, & Heizmann, 1987). Therefore, these cells may be GABAergic interneurons.

**Figure 7:**
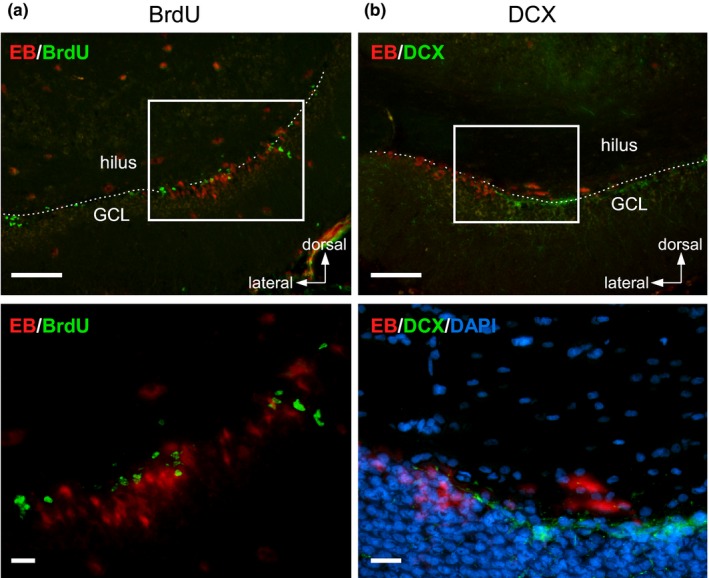
Immunohistochemistry for neurogenic marker molecules in the HIP. (a, b) Photomicrographs showing signals of 5‐bromo‐2′‐deoxyuridine (BrdU, green) (a) and doublecortin (DCX, green) (b), together with EB. Rats were given BrdU (10 mg/kg bw in vehicle) via an implanted atrial catheter once a day for four consecutive days before sacrifice. White broken lines indicate the hilar border of the GCL. Bottom panels magnify the areas enclosed with white squares in the top panels. Cell nuclei were counterstained with DAPI (blue) in the study for DCX. Scale bars, 100 µm (top), 20 µm (bottom)

In the hilar border of the GCL, medium‐sized EB‐positive cells (approximately 10 µm) were negative for BrdU and DCX (Figure 7), suggesting these were not proliferating cells or immature neurons. Furthermore, regarding angiogenesis, few endothelial cells immunoreactive for BrdU were found (data not shown).

## 4 DISCUSSION

Blood‐borne proteins such as albumin and hydrophilic hormones are thought to be unable to leak into most brain areas including the HIP, except for the CVOs, because of the integrity of the BBB in the physiologically normal state. Contrary to the commonly accepted view, several studies have suggested that some hormones can access the HIP. Ghrelin was reported to regulate the synapse density of the HIP (Diano et al., 2006) and to stimulate adult neurogenesis directly (Li et al., 2013). Also, our recent study showed that blood‐borne Ang II stimulated the HIP, thereby enhancing neurogenesis in physiologically normal adult rats (Koyama et al., 2018; Mukuda et al., 2014). Although the penetration of blood‐derived signal molecules into the HIP is plausible, their direct access to hippocampal neurons has not been demonstrated. In the present study, EB was injected intravenously to histologically trace the translocation of the EB‐Alb complex into the HIP of physiologically normal rats. EB binds rapidly to Alb in the blood. Thus, this complex cannot leak into the HIP (Yao et al., 2018) although the translocation of Alb is possible if an endocytotic transport system, including processes containing albondin, is present in the endothelia of the BBB. Together with the leakage of EB in the neuropil of CVOs such as the SFO and ME, we found that hippocampal neurons, especially in the rostral part of the dentate gyrus, entrapped and accumulated EB in their cell bodies in the Ang II and vehicle groups. These findings strongly indicate that blood‐borne products have the potential to access hippocampal neurons directly. Compared with vehicle treatment, greater numbers of neurons did not uptake EB in the HIP even when Ang II was given. However, Ang II treatment led to the neuronal uptake of EB in several regions of the cerebral cortex including the amygdala and retrosplenial cortex, which is physiologically protected by the BBB (Hamasaki et al., unpublished data). These indicate that the neuronal uptake of blood‐borne EB in the HIP occurred constitutively in the physiologically normal state even in the absence of stimuli such as Ang II to increase vascular permeability.

The neuronal uptake of blood‐borne EB is concluded to have occurred within the HIP because the neuronal projection of the EB‐entrapped cells was confined within the HIP. Of the EB‐entrapped neurons, large, oval, or spindle neurons (>15 µm) localized in the hilus and molecular layer were immunopositive for PV, which is exclusively expressed in GABAergic interneurons such as basket cells and axo‐axonic chandelier cells in the hippocampal dentate gyrus (Kosaka et al., 1987). These interneurons form synapses within the dentate gyrus (Booker & Vida, 2018; Freund & Buzsáki, 1996; Pelkey et al., 2017). Large EB‐positive PV‐negative neurons localized along the inner margin of the GCL including the SGZ may be “sporadically lurking Huntingtin‐associated protein 1‐immunoreactive cells,” which are localized in the SGZ and identified as PV‐negative basket cells (Islam et al., 2012).

In addition to these hilar neurons, large EB‐positive PV‐negative neurons in the molecular layer may be molecular layer perforant path‐associated (MOPP) or outer molecular layer (OML) cells, based on their laminar distribution and shape, which had somata in the molecular layer. Axonal networks of MOPPs are restricted to the dentate molecular layer, whereas OMLs project into heavily ramified axons in the molecular layer although some make synapses in the subiculum over the hippocampal fissure (Booker & Vida, 2018). Granule cells project dendrites to the molecular layer to receive inputs from the entorhinal cortex, while the axon forms a mossy fiber in the hilus and transmits signals to pyramidal cells in the CA3. However, all pyramidal neurons in the CA, including CA1 that projects to the entorhinal cortex, were negative for EB. This conclusion is supported by the finding that the neuronal uptake of EB in the HIP was detected within 15 min after injection. This short period did not allow EB to be transported retrogradely from axon terminals in contact with blood in the posterior pituitary to the cell bodies of neuroendocrine cells localized in the PVN and SON. The linear distance from the posterior pituitary to the PVN or SON was simply estimated over 4 mm on a plane of a stereotaxic brain atlas of rats edited by Paxinos and Watson (2009). Taken together, these findings strongly indicate that the EB‐Alb complex reached hippocampal neurons from blood vessels distributed in the HIP or near vicinity in a physiologically normal state. This suggests we should re‐examine the long‐accepted theory that the HIP is impermissible to direct contact with signaling molecules such as Alb and hydrophilic hormones from the systemic circulation under a physiological state. These findings may provide valuable insights to explore the modulation of the hippocampal structure and function by hormones.

The mechanisms involved in how the EB‐Alb complex penetrates into the HIP were not examined in the present study. However, at least three access routes for the EB‐Alb complex might be considered. First, direct leakage of the EB‐Alb complex from blood vessels within the HIP occurred. Although capillaries in the HIP are thought to be impermeable to blood proteins in a physiologically normal state because of the BBB, pathological conditions, such as chronic hypertension and inflammation, cause the disintegration of the BBB, thereby causing the leakage of serum components into the HIP (Biancardi et al., 2014; Ueno et al., 2004; del Valle et al., 2008). In addition, BBB disruption related to increasing age was detected in the HIP of humans (Montagne et al., 2015). Furthermore, in neonatal rats, maternal isolation to induce distress resulted in the leakage of blood‐borne EB into the HIP and other brain regions (Gómez‐González & Escobar, 2009). Ueno et al. (2004) reported the selective fragility of the BBB in the HIP. Accordingly, the present study attempted to increase the vascular permeability of the hippocampal BBB accompanied by the expected structural changes of blood vessels by the daily treatment of Ang II. This peptide increases the permeability of the BBB by stimulating the induction of pores (fenestrae) on endothelial cells (Bodor et al., 2012; Fleegal‐DeMotta et al., 2009) and promoting endothelial cell proliferation for angiogenesis (Amaral, Papanek, & Greene, 2001; Munzenmaier & Greene, 2006). However, few endothelial cells in the HIP were labeled with BrdU administered daily as a single dose, every day for 4 days, before sacrifice in the Ang II and vehicle treatment groups. This suggests that angiogenesis was not induced in the HIP. Initially, we expected that the vascular permeability of the HIP would be increased by elevated Ang II levels in the systemic circulation. However, daily Ang II injection did not change the number of EB‐entrapped neurons in the HIP, although it induced EB‐positive neurons in some parts of the neocortex such as the amygdala and retrosplenial cortex (Hamasaki et al., unpublished data). We found EB‐entrapped neurons in the HIP of the vehicle group, indicating the constitutive access of blood‐borne EB to the HIP in the absence of stimuli to increase the BBB permeability. Taken together, stimulus‐independent leaky blood vessels may be localized in the HIP, which physiologically allow blood‐borne products direct access to the HIP. A detailed examination of the ultrastructure of blood vessels in the HIP is needed.

The second route may consist of a pathway from the SFO to the HIP. Ueno and colleagues used several strains of mice including a pathological model to demonstrate that an exogenously injected plant hemoprotein HRP penetrated into the parenchyma of the SFO from leaky blood vessels and then diffused to the HIP, especially the rostromedial region (Ueno et al., 1994, 1997, 2000). The SFO is a member of the CVOs and is localized immediately rostral to the HIP in rodents and allows hormones to transmit systemic signals to the brain (Fitzsimons, 1998). The hippocampal diffusion of HRP shown in mice is consistent with the dominant distribution area of EB‐accumulating neurons in the present study. For blood‐borne products, a similar pathway connecting the CVOs and the brain area inside the BBB was reported in the hypothalamus. The hormone ghrelin invaded the ME from leaky blood vessels and then diffused into the Arc inside the BBB to activate neurons (Schaeffer et al., 2013). The present study also confirmed the ME‐Arc axis by identifying the neuronal uptake of blood‐borne EB in the Arc. This suggests an SFO‐HIP axis might exist in addition to the ME‐Arc axis in rats. This route would be available for blood‐borne products to access to HIP constitutively without the influence of stimuli to increase vascular permeability because the SFO is always open. The third route might be possible when the specifically controlled transcellular transport system for Alb is present in the endothelia of the hippocampal BBB. Several studies using cultured or isolated pulmonary microvessels demonstrated that Alb‐binding proteins, such as albondin expressed on the endothelia, facilitated transcytosis (Merlot, Kalinowski, & Richardson, 2014; Shasby & Peterson, 1987; Tiruppathi, Song, Bergenfeldt, Sass, & Malik, 1997; Vogel, Minshall, Pilipović, Tiruppathi, & Malik, 2001). However, Alb transport into the brain via this pathway might be restricted because of the low or lack of expression of albondin in brain‐derived microvessels (Schnitzer, 1992). However, neonatal fragment crystallizable receptor (FcRn), which binds to IgG, was reported to provide a binding site for Alb and be expressed in the BBB endothelia (Pyzik et al., 2019). FcRn mediates transcytosis across the BBB not only from the brain to the blood, but also from the blood to the brain (Pyzik et al., 2019; St‐Amour et al., 2013). However, the trafficking behavior mediated by FcRn has not been examined in the HIP.

As seen in traumatic injury or neuroinflammation, pathologically induced BBB disruption causes a large amount of leakage of EB from blood vessels; therefore, these brain regions are easily distinguished macroscopically by blue staining or by intense red fluorescence under fluorescent microscopy (Alonso et al., 2011; Rákos et al., 2007; Roe et al., 2012; Yang et al., 2007). Accordingly, many studies using fluorescent microscopy have attempted to detect EB remaining in the extracellular space of the brain parenchyma. However, it is difficult to detect low EB signals using such conventional analyses because of the poor S/N ratio. Therefore, in some cases, the low extravasation of EB may have been missed. However, even if levels of EB are too low to be observed in the extracellular space by histological methods, it can be entrapped in living neurons through endocytosis and accumulate in cell bodies, thereby emitting distinguishably intensified fluorescence (Albanese & Bentivoglio, 1982; Mukuda & Ando, 2003, 2010). To the best of our knowledge, the neuronal uptake of EB in the HIP was shown only in a pathological model of temporal lobe epilepsy, where hippocampal hyperactivity was elicited by an intrahippocampal administration of kainic acid (Liu, Liu, Wang, Liu, & Zhao, 2016). Thus, the present study is the first to successfully identify a small amount of blood‐borne EB, which could not be distinguished using conventional methods, in physiologically normal rats by detecting dye accumulated in the cell bodies of hippocampal neurons. This methodology may be advantageous for detecting very small amounts of probes entrapped by neurons although it may depend on the efficiency of neuronal uptake.

In addition to the neuronal uptake of EB in hilar interneurons, some granule cells in the hilar boundary area, probably including the SGZ, were positive for EB. The SGZ contains neuronal stem cells throughout life and maintains neurogenesis even in adulthood, in which new neurons are incorporated into the GCL to form neuronal networks (Lacefield, Itskov, Reardon, Hen, & Gordon, 2012). An immunohistochemical assessment using BrdU and DCX was performed to examine whether these EB‐positive neurons were new neurons. However, EB‐positive neurons were not immunoreactive for BrdU or DCX. This suggests that these cells were not proliferating and therefore were at least 2 weeks or older because DCX is expressed in new neurons within 2 weeks after birth. Further examination of these granule cells and the neurochemical identification of neurons positive for EB in the HIP are needed to elucidate the mechanisms involved in how these neurons exclusively entrap EB.

With the progression of experimental research using model animals, the HIP has been suggested to be susceptible to blood‐borne molecules, such as Ang II and ghrelin, which enhanced hippocampal neurogenesis through the direct activation of hippocampal cells (Koyama et al., 2018; Li et al., 2013; Mukuda et al., 2014). However, the access routes of hormones have not been fully determined. An exogenously administrated neurotoxicant organotin compound, trimethyltin chloride (TMT), induced the selective neuronal cell death of granule cells and CA3 pyramidal neurons in the rostromedial portion of the HIP in rats (Chang & Dyer, 1983; Latini et al., 2010). Although TMT is thought to cross the BBB, a definitive explanation on the vulnerability of the HIP to TMT has not been obtained. Constitutive access of the HIP by blood‐borne components may allow more TMT in the blood to enter into the HIP, compared with other brain regions. Furthermore, an animal model of temporal lobe epilepsy suggested that the direct action of Alb in the HIP was a trigger for epileptogenesis (Weissberg, Reichert, Heinemann, & Friedman, 2011). This phenomenon can be explained by the disruption of the BBB after brain injury. However, the mechanisms associated with the change from a normally functioning brain to an epileptic state have not been fully elucidated. Diffusion from the SFO or direct leakage from the blood vessels of a fragile hippocampal BBB may allow blood‐borne contents access to the HIP selectively, compared with other brain regions. Our findings of the constitutive access of blood‐derived proteins into the HIP provide a useful insight to elucidate the mechanisms of various physiological and pathogenic interactions between the blood and brain.

## CONFLICT OF INTEREST

Dr. Mukuda, Dr. Koyama, Dr. Nakane, and Prof. Kaidoh declare no conflicts of interest.

## AUTHOR CONTRIBUTIONS

S.H. and T.M. designed the research; S.H., T.M., Y.K., H.N., and T.K. performed the experiments and interpreted the results of experiments; and S.H. and T.M. analyzed the data, prepared figures, and drafted the manuscript.

## Data Availability

The data that support the findings of this study are available from the corresponding author upon reasonable request.

## References

[brb31544-bib-0001] Albanese, A. , & Bentivoglio, M. (1982). Retrograde fluorescent neuronal tracing combined with acetylcholinesterase histochemistry. Journal of Neuroscience Methods, 6, 121–127. 10.1016/0165-0270(82)90022-X 7121056

[brb31544-bib-0002] Alonso, A. , Reinz, E. , Fatar, M. , Hennerici, M. G. , & Meairs, S. (2011). Clearance of albumin following ultrasound‐induced blood‐brain barrier opening is mediated by glial but not neuronal cells. Brain Research, 1411, 9–16. 10.1016/j.brainres.2011.07.006 21820103

[brb31544-bib-0003] Amaral, S. L. , Papanek, P. E. , & Greene, A. S. (2001). Angiotensin II and VEGF are involved in angiogenesis induced by short‐term exercise training. The American Journal of Physiology‐Heart and Circulatory Physiology, 281, H1163–H1169. 10.1152/ajpheart.2001.281.3.H1163 11514283

[brb31544-bib-0004] Banks, W. A. , Tschöp, M. , Robinson, S. M. , & Heiman, M. L. (2002). Extent and direction of ghrelin transport across the blood‐brain barrier is determined by its unique primary structure. Journal of Pharmacology and Experimental Therapeutics, 302, 822–827. 10.1124/jpet.102.034827 12130749

[brb31544-bib-0005] Biancardi, V. C. , Son, S. J. , Ahmadi, S. , Filosa, J. A. , & Stern, J. E. (2014). Circulating angiotensin II gains access to the hypothalamus and brain stem during hypertension via breakdown of the blood‐brain barrier. Hypertension, 63, 572–579. 10.1161/HYPERTENSIONAHA.113.01743 24343120 PMC4080808

[brb31544-bib-0006] Bodor, C. , Nagy, J. P. , Végh, B. , Németh, A. , Jenei, A. , MirzaHosseini, S. , … Rosivall, L. (2012). Angiotensin II increases the permeability and PV‐1 expression of endothelial cells. The American Journal of Physiology‐Cell Physiology, 302, C267–276. 10.1152/ajpcell.00138.2011 22012329

[brb31544-bib-0007] Booker, S. A. , & Vida, I. (2018). Morphological diversity and connectivity of hippocampal interneurons. Cell and Tissue Research, 373, 619–641. 10.1007/s00441-018-2882-2 30084021 PMC6132631

[brb31544-bib-0008] Chang, L. W. , & Dyer, R. S. (1983). A time‐course study of trimethyltin induced neuropathology in rats. Neurobehavioral Toxicology and Teratology, 5, 443–459. 6646318

[brb31544-bib-0009] del Valle, J. , Camins, A. , Pallàs, M. , Vilaplana, J. , & Pelegrí, C. (2008). A new method for determining blood‐brain barrier integrity based on intracardiac perfusion of an Evans Blue‐Hoechst cocktail. Journal of Neuroscience Methods, 174, 42–49. 10.1016/j.jneumeth 18647619

[brb31544-bib-0010] Diano, S. , Farr, S. A. , Benoit, S. C. , McNay, E. C. , da Silva, I. , Horvath, B. , … Horvath, T. L. (2006). Ghrelin controls hippocampal spine synapse density and memory performance. Nature Neuroscience, 9, 381–388. 10.1038/s41366-018-0126-x 16491079

[brb31544-bib-0011] Fitzsimons, J. T. (1998). Angiotensin, thirst, and sodium appetite. Physiological Reviews, 78, 583–686. 10.1152/physrev.1998.78.3.583 9674690

[brb31544-bib-0012] Fleegal‐DeMotta, M. A. , Doghu, S. , & Banks, W. A. (2009). Angiotensin II modulates BBB permeability via activation of the AT_1_ receptor in brain endothelial cells. Journal of Cerebral Blood Flow & Metabolism, 29, 640–647. 10.1038/jcbfm.2008.158 19127280

[brb31544-bib-0013] Freund, T. F. , & Buzsáki, G. (1996). Interneurons of the hippocampus. Hippocampus, 6, 347–470. 10.1002/(SICI)1098-1063(1996)6:4<347:AID-HIPO1>3.0.CO;2-I 8915675

[brb31544-bib-0014] Gómez‐González, B. , & Escobar, A. (2009). Altered functional development of the blood‐brain barrier after early life stress in the rat. Brain Research Bulletin, 79, 376–387. 10.1016/j.brainresbull.2009.05.012 19463912

[brb31544-bib-0015] Islam, M. N. , Fujinaga, R. , Yanai, A. , Jahan, M. R. , Takeshita, Y. , Kokubu, K. , & Shinoda, K. (2012). Characterization of the “sporadically lurking HAP1‐immunoreactive (SLH) cells” in the hippocampus, with special reference to the expression of steroid receptors, GABA, and progenitor cell markers. Neuroscience, 210, 67–81. 10.1016/j.neuroscience.2012.02.029 22421101

[brb31544-bib-0016] Kosaka, T. , Katsumaru, H. , Hama, K. , Wu, J. Y. , & Heizmann, C. W. (1987). GABAergic neurons containing the Ca^2+^‐binding protein parvalbumin in the rat hippocampus and dentate gyrus. Brain Research, 419, 119–130. 10.1016/0006-8993(87)90575-0 3315112

[brb31544-bib-0017] Koyama, Y. , Mukuda, T. , Hamasaki, S. , Nakane, H. , & Kaidoh, T. (2018). Short‐term heat exposure promotes hippocampal neurogenesis via activation of angiotensin II type 1 receptor in adult rats. Neuroscience, 385, 121–132. 10.1016/j.neuroscience.2018.05.045 29902505

[brb31544-bib-0018] Lacefield, C. O. , Itskov, V. , Reardon, T. , Hen, R. , & Gordon, J. A. (2012). Effects of adult‐generated granule cells on coordinated network activity in the dentate gyrus. Hippocampus, 22, 106–116. 10.1002/hipo.20860 20882540 PMC3282563

[brb31544-bib-0019] Latini, L. , Geloso, M. C. , Corvino, V. , Giannetti, S. , Florenzano, F. , Viscomi, M. T. , … Molinari, M. (2010). Trimethyltin intoxication up‐regulates nitric oxide synthase in neurons and purinergic ionotropic receptor 2 in astrocytes in the hippocampus. Journal of Neuroscience Research, 88, 500–509. 10.1002/jnr.22238 19795376

[brb31544-bib-0020] Li, E. , Chung, H. , Kim, Y. , Kim, D. H. , Ryu, J. H. , Sato, T. , … Park, S. (2013). Ghrelin directly stimulates adult hippocampal neurogenesis: Implications for learning and memory. Endocrine Journal, 60, 781–789. 10.1507/endocrj.EJ13-0008 23411585

[brb31544-bib-0021] Li, Z. , Cao, Y. , Li, L. , Liang, Y. , Tian, X. , Mo, N. , … Guo, X. (2014). Prophylactic angiotensin type 1 receptor antagonism confers neuroprotection in an aged rat model of postoperative cognitive dysfunction. Biochemical and Biophysical Research Communications, 449, 74–80. 10.1016/j.bbrc.2014.04.153 24814703

[brb31544-bib-0022] Li, Z. , Mo, N. , Li, L. , Cao, Y. , Wang, W. , Liang, Y. , … Guo, X. (2016). Surgery‐induced hippocampal angiotensin II elevation causes blood‐brain barrier disruption via MMP/TIMP in aged rats. Frontiers in Cellular Neuroscience, 10, 105 10.3389/fncel.2016.00105 27199659 PMC4844612

[brb31544-bib-0023] Liu, Z. , Liu, J. , Wang, S. , Liu, S. , & Zhao, Y. (2016). Neuronal uptake of serum albumin is associated with neuron damage during the development of epilepsy. Experimental and Therapeutic Medicine, 12, 695–701. 10.3892/etm.2016.3397 27446263 PMC4950244

[brb31544-bib-0024] Merlot, A. M. , Kalinowski, D. S. , & Richardson, D. R. (2014). Unraveling the mysteries of serum albumin‐more than just a serum protein. Frontiers in Physiology, 12(5), 299 10.3389/fphys.2014.00299 PMC4129365 25161624

[brb31544-bib-0025] Montagne, A. , Barnes, S. R. , Sweeney, M. D. , Halliday, M. R. , Sagare, A. P. , Zhao, Z. , … Zlokovic, B. V. (2015). Blood‐brain barrier breakdown in the aging human hippocampus. Neuron, 85, 296–302. 10.1016/j.neuron.2014.12.032 25611508 PMC4350773

[brb31544-bib-0026] Mukuda, T. , & Ando, M. (2003). Medullary motor neurones associated with drinking behaviour of Japanese eels. The Journal of Fish Biology, 62, 1–12. 10.1046/j.1095-8649.2003.00002.x

[brb31544-bib-0027] Mukuda, T. , & Ando, M. (2010). Central regulation of the pharyngeal and upper esophageal reflexes during swallowing in the Japanese eel. The Journal of Comparative Physiology A, 196, 111–122. 10.1007/s00359-009-0498-4 20035336

[brb31544-bib-0028] Mukuda, T. , Koyama, Y. , Hamasaki, S. , Kaidoh, T. , & Furukawa, Y. (2014). Systemic angiotensin II and exercise‐induced neurogenesis in adult rat hippocampus. Brain Research, 1588, 92–103. 10.1016/j.brainres.2014.09.019 25223907

[brb31544-bib-0029] Munzenmaier, D. H. , & Greene, A. S. (2006). Chronic angiotensin II AT1 receptor blockade increases cerebral cortical microvessel density. The American Journal of Physiology‐Heart and Circulatory Physiology, 290, H512–H516. 10.1152/ajpheart.01136.2004 16199473

[brb31544-bib-0030] Paxinos, G. , & Watson, C. (2009). The rat brain in stereotaxic coordinates, compact (6th ed.). Amsterdam, The Netherlands: Elsevier.

[brb31544-bib-0031] Pelkey, K. A. , Chittajallu, R. , Craig, M. T. , Tricoire, L. , Wester, J. C. , & McBain, C. J. (2017). Hippocampal GABAergic inhibitory interneurons. Physiological Reviews, 97, 1619–1747. 10.1152/physrev.00007.2017 28954853 PMC6151493

[brb31544-bib-0032] Pyzik, M. , Sand, K. M. K. , Hubbard, J. J. , Andersen, J. T. , Sandlie, I. , & Blumberg, R. S. (2019). The neonatal Fc receptor (FcRn): A misnomer? Frontiers in Immunology, 10, 1540 10.3389/fimmu.2019.01540 31354709 PMC6636548

[brb31544-bib-0033] Rákos, G. , Kis, Z. , Nagy, D. , Lür, G. , Farkas, T. , Hortobágyi, T. , … Toldi, J. (2007). Evans Blue fluorescence permits the rapid visualization of non‐intact cells in the perilesional rim of cold‐injured rat brain. Acta Neurobiologiae Experimentalis, 67, 149–154. 17691222 10.55782/ane-2007-1642

[brb31544-bib-0034] Rhea, E. M. , Salameh, T. S. , Gray, S. , Niu, J. , Banks, W. A. , & Tong, J. (2018). Ghrelin transport across the blood‐brain barrier can occur independently of the growth hormone secretagogue receptor. Molecular Metabolism, 18, 88–96. 10.1016/j.molmet.2018.09.007 30293893 PMC6308033

[brb31544-bib-0035] Roe, K. , Kumar, M. , Lum, S. , Orillo, B. , Nerurkar, V. R. , & Verma, S. (2012). West Nile virus‐induced disruption of the blood‐brain barrier in mice is characterized by the degradation of the junctional complex proteins and increase in multiple matrix metalloproteinases. Journal of General Virology, 93, 1193–1203. 10.1099/vir.0.040899-0 22398316 PMC3755517

[brb31544-bib-0036] Schaeffer, M. , Langlet, F. , Lafont, C. , Molino, F. , Hodson, D. J. , Roux, T. , … Mollard, P. (2013). Rapid sensing of circulating ghrelin by hypothalamic appetite‐modifying neurons. Proceedings of the National Academy of Sciences of the United States of America, 110, 1512–1517. 10.1073/pnas.1212137110 23297228 PMC3557016

[brb31544-bib-0037] Schnitzer, J. E. (1992). gp60 is an albumin‐binding glycoprotein expressed by continuous endothelium involved in albumin transcytosis. American Journal of Physiology, 262, H246–H254. 10.1152/ajpheart.1992.262.1.H246 1733316

[brb31544-bib-0038] Shasby, D. M. , & Peterson, M. W. (1987). Effects of albumin concentration on endothelial albumin transport *in vitro* . American Journal of Physiology, 253, H654–H661. 10.1152/ajpheart.1987.253.3.H654 2443023

[brb31544-bib-0039] St‐Amour, I. , Paré, I. , Alata, W. , Coulombe, K. , Ringuette‐Goulet, C. , Drouin‐Ouellet, J. , … Calon, F. (2013). Brain bioavailability of human intravenous immunoglobulin and its transport through the murine blood‐brain barrier. Journal of Cerebral Blood Flow & Metabolism, 33, 1983–1992. 10.1038/jcbfm.2013.160 24045402 PMC3851908

[brb31544-bib-0040] Tiruppathi, C. , Song, W. , Bergenfeldt, M. , Sass, P. , & Malik, A. B. (1997). Gp60 activation mediates albumin transcytosis in endothelial cells by tyrosine kinase‐dependent pathway. Journal of Biological Chemistry, 272, 25968–25975. 10.1074/jbc.272.41.25968 9325331

[brb31544-bib-0041] Ueno, M. , Akiguchi, I. , Hosokawa, M. , Kotani, H. , Kanenishi, K. , & Sakamoto, H. (2000). Blood‐brain barrier permeability in the periventricular areas of the normal mouse brain. Acta Neuropathologica, 99, 385–392. 10.1007/s004010051140 10787037

[brb31544-bib-0042] Ueno, M. , Akiguchi, I. , Hosokawa, M. , Shinnou, M. , Sakamoto, H. , Takemura, M. , & Higuchi, K. (1997). Age‐related changes in the brain transfer of blood‐borne horseradish peroxidase in the hippocampus of senescence‐accelerated mouse. Acta Neuropathologica, 93, 233–240. 10.1007/s004010050609 9083554

[brb31544-bib-0043] Ueno, M. , Akiguchi, I. , Hosokawa, M. , Yagi, H. , Takemura, M. , Kimura, J. , & Takeda, T. (1994). Accumulation of blood‐borne horseradish peroxidase in medial portions of the mouse hippocampus. Acta Neurologica Scandinavica, 90, 400–404. 10.1111/j.1600-0404.1994.tb02748.x 7892758

[brb31544-bib-0044] Ueno, M. , Sakamoto, H. , Tomimoto, H. , Akiguchi, I. , Onodera, M. , Huang, C. L. , & Kanenishi, K. (2004). Blood‐brain barrier is impaired in the hippocampus of young adult spontaneously hypertensive rats. Acta Neuropathologica, 107, 532–538. 10.1007/s00401-004-0845-z 15042385

[brb31544-bib-0045] Uyama, O. , Okamura, N. , Yanase, M. , Narita, M. , Kawabata, K. , & Sugita, M. (1988). Quantitative evaluation of vascular permeability in the gerbil brain after transient ischemia using Evans blue fluorescence. Journal of Cerebral Blood Flow & Metabolism, 8, 282–284. 10.1038/jcbfm.1988.59 3343300

[brb31544-bib-0046] Vogel, S. M. , Minshall, R. D. , Pilipović, M. , Tiruppathi, C. , & Malik, A. B. (2001). Albumin uptake and transcytosis in endothelial cells in vivo induced by albumin‐binding protein. American Journal of Physiology, 281, L1512–L1522. 10.1152/ajplung.2001.281.6.L1512 11704548

[brb31544-bib-0047] Weissberg, I. , Reichert, A. , Heinemann, U. , & Friedman, A. (2011). Blood‐brain barrier dysfunction in epileptogenesis of the temporal lobe. Epilepsy Research and Treatment, 2011, 143908 10.1155/2011/143908 22937228 PMC3420538

[brb31544-bib-0048] Yang, F. Y. , Fu, W. M. , Yang, R. S. , Liou, H. C. , Kang, K. H. , & Lin, W. L. (2007). Quantitative evaluation of focused ultrasound with a contrast agent on blood‐brain barrier disruption. Ultrasound in Medicine and Biology, 33, 1421–1427. 10.1016/j.ultrasmedbio.2007.04.006 17561334

[brb31544-bib-0049] Yao, L. , Xue, X. , Yu, P. , Ni, Y. , & Chen, F. (2018). Evans blue dye: A revisit of its applications in biomedicine. Contrast Media & Molecular Imaging, 22(2018), 7628037 10.1155/2018/7628037 PMC5937594 29849513

